# Incorporating prognosis in the care of older adults with multimorbidity: description and evaluation of a novel curriculum

**DOI:** 10.1186/s12909-015-0488-x

**Published:** 2015-12-01

**Authors:** Nancy L. Schoenborn, Cynthia Boyd, Danelle Cayea, Kelly Nakamura, Qian-Li Xue, Anushree Ray, Matthew McNabney

**Affiliations:** Division of Geriatric Medicine and Gerontology, Department of Medicine, Johns Hopkins University School of Medicine, 5200 Eastern Avenue, Mason F. Lord Building Center Tower, Suite 2200, Baltimore, MD 21224 USA; Harborview Medical Center, University of Washington, Seattle, WA USA; Medline Industries, Mundelein, IL USA

**Keywords:** Prognosis, Multimorbidity, Older adults, Resident education, Curriculum, Primary care

## Abstract

**Background:**

Prognosis is a critical consideration in caring for older adults with multiple chronic conditions, or “multimorbidity”. Clinicians are not adequately trained in this area. We describe an innovative curriculum that teaches internal medicine residents how to incorporate prognosis in the care of older adults with multimorbidity.

**Methods:**

The curriculum includes three small-group sessions and a clinical exercise; it focuses on the assessment, communication, and application of prognosis to inform clinical decisions. The curriculum was implemented with 20 first-year residents at one university-based residency (intervention group). Fifty-two first-year residents from a separate residency affiliated with the same university served as controls.

Evaluation included three components. A survey assessed acceptability. A pre/post survey assessed attitude, knowledge, and self-reported skills (Impact survey). Comparison of baseline and follow-up results used paired t-test and McNemar test; comparison of inter-group differences used t-test and Fisher’s exact test. A retrospective, blinded pre/post chart review assessed documentation behavior; abstracted outcomes were analyzed using Fisher’s exact test.

**Results:**

The curriculum was highly rated (4.5 on 5-point scale). Eighteen intervention group residents (90 %) and 29 control group residents (56 %) responded to the Impact survey. At baseline, there were no significant inter-group differences in any of the responses. The intervention group improved significantly in prognosis communication skills (5.2 to 6.6 on 9-point scale, *p* < 0.001), usage of evidence-based prognostic tools (1/18 to 14/18 responses, *p* < 0.001), and prognostic accuracy (1/18 to 9/18 responses, *p* = 0.005). These responses were significantly different from the control group at follow-up.

Of 71 charts reviewed in each group, prognosis documentation in the intervention group increased from 1/25 charts (4 %) at baseline to 8/46 charts (17 %) at follow-up (*p* = 0.15). No prognosis documentation was identified in the control group at either time point. Inter-group difference was significant at follow-up (*p* = 0.006).

**Conclusion:**

We developed and implemented a novel prognosis curriculum that had significant short-term impact on the residents’ knowledge and communication skills as compared to a control group. This innovative curriculum addresses an important educational gap in incorporating prognosis in the care of older adults with multimorbidity.

**Electronic supplementary material:**

The online version of this article (doi:10.1186/s12909-015-0488-x) contains supplementary material, which is available to authorized users.

## Background

Older adults often have multiple chronic conditions, or multimorbidity, which is associated with increased morbidity and mortality [1–3]. In this heterogeneous population, prognostication that takes into account comorbidities and functional status helps differentiate older adults in relatively good health and those with more limited prognosis. Research and clinical practice recommendations increasingly advocate for incorporating prognosis to individualize care decisions that have long lag-time to benefit but potential short-term harms, such as cancer screening and diabetes glycemic control, in the care of older adults [1, 4–13].

Prognosis is not often incorporated in clinical decisions; [14–19] a major contributor may be inadequate clinician training [20]. Existing curricula on prognosis are limited to palliative care or oncology; [21–26] we have not found any described curriculum that teaches assessing, discussing, and incorporating prognosis to frame clinical decisions in the care of older adults with multimorbidity.

Internal medicine residents are a key learner group because residency is a formative time and many older adults receive care from clinicians trained in internal medicine [27]. Assessing and incorporating prognosis to inform care are part of key competencies for internal medicine residents [28]. We describe the development and evaluation of a novel curriculum that teaches internal medicine residents how to incorporate prognosis to inform clinical decisions in primary care of older adults with multimorbidity.

## Methods

### Curriculum description

The curriculum was developed during a year-long curriculum development course and all materials were developed in an iterative fashion based on feedback from course leaders and participants. Curriculum development used a structured approach and drew from Kolb’s model of experiential learning [29, 30]. The learner objectives and the corresponding evaluation strategies are outlined in Table 1. The curriculum includes three small-group sessions and a clinical exercise. The curriculum focuses on life expectancy but also teaches about condition-specific prognosis or risk (e.g. risk of stroke from atrial fibrillation). Session 1 introduces the importance of prognosis, uses case-based exercises to teach prognostic tools and how to apply prognosis to frame the benefits/harms of common clinical decisions in primary care. A summary of prognostic tools and resources is provided to residents to facilitate application (Additional file 1). Session 2 uses role play exercises with standardized patients to teach the skill to communicate the benefits/harms of a decision that is framed by prognosis and the skill to discuss prognosis explicitly. In the clinical exercise, residents assess prognosis for one of their primary care patients, use the estimated prognosis to frame a relevant clinical decision, and have a discussion with the patient either about the clinical decision or explicitly about the patient’s prognosis. During Session 3, the residents share their reflections about the exercise and receive group feedback.

**Table 1:** Learner objectives and corresponding evaluation strategies

Objectives	Evaluation strategies
Acceptability	- Post-only acceptability survey of the Intervention group
- Rate the curriculum as acceptable and relevant.	
Attitude	- Pre/post survey (Impact Survey) of Intervention and Control groups
- Rate incorporating prognosis to inform clinical decisions as important in the care of older adults with multimorbidity.	
Knowledge/Skills	- Pre/post survey (Impact Survey) of Intervention and Control groups
- Demonstrate assessment of prognosis using evidence-based tools.	
- Demonstrate application of prognosis to inform clinical decisions.	
- Be able to communicate prognosis a) as incorporated into the benefits/harms discussion related to a clinical decision, and b) explicitly in a discussion	
Behavior	- Pre/post chart review of Intervention and Control groups for prognosis documentation
- Routinely incorporate prognosis to inform clinical decisions in the care of older adults with multimorbidity.	

### Curriculum implementation

There are two internal medicine residency programs at our university; neither program had any pre-existing curricula on prognosis. The prognosis curriculum was first implemented in January 2014 in one residency program with 20 first-year residents (intervention group) while the other program’s 52 first-year residents served as controls. The curriculum was implemented in an existing, required rotation that consisted of two 2-week outpatient blocks.

### Curriculum assessment

Evaluation was informed by the Miller’s pyramid of clinical assessment; [31] it consisted of three strategies: a survey to assess acceptability, a pre/post survey to assess impact on attitude, knowledge, and self-reported skills, and a pre/post chart review to assess impact on documentation behavior (Table 1). This study was approved by an institutional review board.

### Evaluation of acceptability

A survey on curriculum acceptability was administered immediately after the curriculum to the intervention group, asking the residents to rate on a 5-point Likert scale (1 = poor, 5 = excellent) the curriculum’s quality and relevance. The results are presented using descriptive statistics.

### Evaluation of attitude, knowledge, skills

A second survey (Impact Survey) was administered at baseline and at follow-up after the curriculum to both groups to assess the curriculum’s impact on attitude, knowledge, and self-reported skills. We did not find any existing validated assessment tools and developed our own instrument; we piloted it for clarity, face and content validity with six geriatric medicine fellows at our institution. We used the same questions at baseline and at follow-up.

Questions on attitude and self-reported skills used 9-point Likert scales (1 = “not at all important”/ “not at all prepared”, 9 = “extremely important”/ “extremely prepared”). Questions on knowledge used two hypothetical patient cases involving older adults with multimorbidity and functional dependence. The respondents were asked to estimate the patients’ 4- or 5-year mortality risk (in percentages) based on the provided clinical information; this was then compared to validated prognostic tools to determine prognostic accuracy [32, 33]. The respondents were also asked to report how they derived the estimate (using a prognostic tool or citing literature versus using clinical experience alone) and apply the estimated prognosis to answer multiple-choice questions about clinical decisions such as screening colonoscopy and glycemic control in older adults with diabetes mellitus.

Comparisons of baseline and follow-up results used paired t-test and McNemar test. Comparisons between intervention and control groups used t-test and Fisher’s exact test. Statistical analyses were performed using STATA 12.0 (StataCorp LP, College Station, TX) and Microsoft Excel 2010 (Microsoft, Redmond, WA).

### Evaluation of behavior

To assess the curriculum’s impact on clinical practice, we conducted a retrospective, controlled, blinded chart review at baseline before the curriculum (September-December 2013) and at follow-up after the curriculum (late February-June 2014). A “chart” is defined as the documentation of a single clinic visit. Charts were eligible if the visit was between a resident in either study group and the resident’s primary care patient who was at least 60 year old and had at least two of 30 conditions in the Elixhauser comorbidity index [34].

In the baseline period, we randomly selected 25 out of 43 eligible charts in the intervention group and 25 out of 128 eligible charts in the control group for analysis. In the follow-up period, we included all 46 eligible charts in the intervention group and randomly selected 46 out of 203 eligible charts in the control group for analysis. A chart abstraction guide assessed for documentation of overall prognosis, which was defined to include: “life expectancy”, “prognosis”, “mortality”, “mortality rate”, “likelihood to live/likelihood to die”, “survival”, “life span”, and “long-term outcome”. If overall prognosis documentation was present, we examined if the prognosis estimate was qualitative (e.g. “good”, “poor”) or quantitative (e.g. “10-year mortality risk is 93 %”), whether any literature or evidence-based tool was cited in deriving the prognosis, whether the prognosis was communicated, and whether it affected a clinical decision. We also examined whether overall prognosis was documented when there was documentation of cancer screening because clinical practice recommendations increasingly advocate for incorporating life expectancy in cancer screening [10–13].

Patient, resident, clinic identifiers and date were removed from charts. Two investigators (NS, KN), blinded to the timing of the visit and the resident’s group designation, reviewed the de-identified charts. We piloted the abstraction guide with 12 charts for clarity, face and content validity, and inter-rater reliability. We revised the abstraction guide with input from three investigators (NS, KN, CB), and used the revised guide to review the remaining charts. Inter-rater agreement was 100 % regarding whether prognosis was documented, whether the estimate was qualitative or quantitative, whether prognostic tool was cited, and whether prognosis was communicated. Inter-rater agreement was 89.9 % regarding whether prognosis affected a clinical decision. Abstracted outcomes were analyzed using Fisher’s exact test. Patient characteristics including demographic information, number of chronic conditions, and number of medications were also abstracted and compared between the intervention and control groups using t-test and Fisher’s exact test. All analyses were conducted using STATA 12.0 (StataCorp LP, College Station, TX) and Microsoft Excel 2010 (Microsoft, Redmond, WA).

## Results

The intervention group included 20 first-year residents (12 women, eight men). The control group included 52 first-year residents (24 women, 28 men).

### Evaluation of acceptability

All 20 intervention group residents completed the survey on curriculum acceptability. On a 5-point Likert scale (1 = poor, 5 = excellent), the residents rated the curriculum highly (mean 4.5, SD 0.5) and found it highly relevant (mean 4.5, SD 0.7).

### Evaluation of attitude, knowledge, skills

Eighteen residents (11 women, seven men) in the intervention group and 29 residents (14 women, 15 men) in the control group responded to the Impact survey at both time points (response rates 90 and 56 % respectively); there were two incomplete responses in the control group. Results are summarized in Table 2.

**Table 2:**
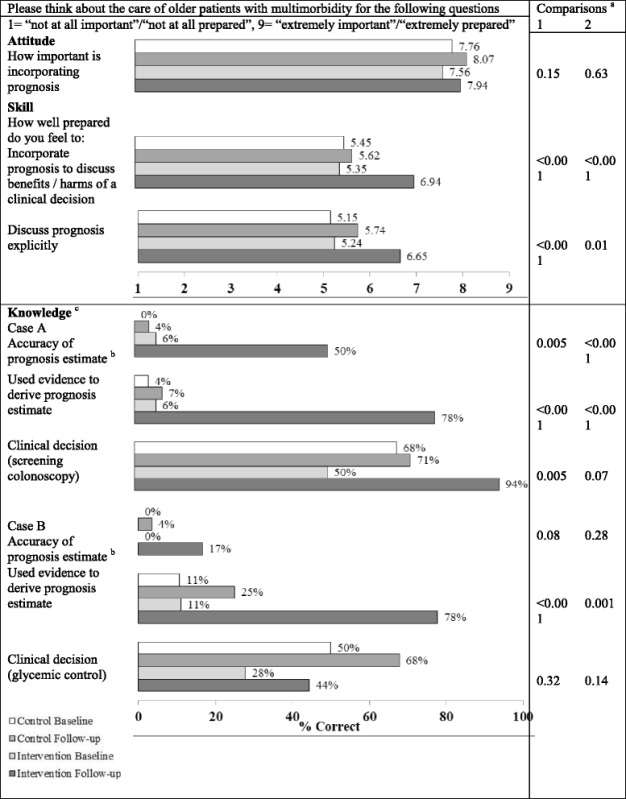
Assessment of the curriculum’s impact on attitude, knowledge, and self-reported skills, comparing intervention group at baseline vs. at follow-up and intervention group vs. control group at follow-up

^a^Comparison 1 is between intervention group at baseline vs. at follow-up. Comparison 2 is between intervention group and control group at follow-up

^b^Prognosis estimates (mortality risk in percentages) were compared to the estimates derived using the Lee index and the Schonberg index [32, 33]

There were no significant differences between the two groups’ responses to any of the survey questions at baseline. The intervention group respondents rated incorporating prognosis as highly important at baseline and at follow-up (7.6 to 7.9 on a 9-point scale, *p* = 0.15). Two aspects of prognosis communication were assessed; self-reported skill levels in both aspects increased significantly from 5.3 to 6.9 and 5.2 to 6.6 on a 9-point scale (both *p* < 0.001); these responses were significantly different from the control group at follow-up.

In knowledge assessment, the intervention group respondents improved in the accuracy of prognosis estimates (i.e. same percentage as predicted by validated tools) in both hypothetical patient cases, reaching statistical significance in one of the two cases (1/18 to 9/18 correct responses, *p* < 0.001). The intervention group respondents also significantly increased in usage of evidence-based prognostication methods in both cases (1/18 to 14/18 and 2/18 to 14/18, both *p* < 0.001); these were significantly different from the control group at follow-up. In both cases, the intervention group respondents improved in correctly answering clinical decision questions that involved applying prognosis information, reaching statistical significance in one of two cases (9/18 to 17/18, *p* = 0.005) but inter-group difference was not significant at follow-up (*p* = 0.07).

### Evaluation of behavior

Chart review results are summarized in Table 3. The intervention group charts involved 15 residents and 49 patients. Most of these patients were male and most were white. The control group charts involved 33 residents and 63 patients. Most of these patients were female and most were African American.

**Table 3:** Chart review results-- patient characteristics and prognosis documentation in the intervention and control groups

		Intervention	Control	*P*-value
Patient characteristics				
Number of patients involved in reviewed charts		*N* = 49	*N* = 63	
Patient age, mean (SD), year		68.9 (7.7)	70.0 (8.3)	0.58
Female patient, number (%)		23 (47 %)	42 (67 %)	0.05
Patient race, number (%)				
- White		31 (63 %)	2 (3 %)	<0.001
- African American		12 (24 %)	56 (89 %)	<0.001
- Other		6 (12 %)	5 (8 %)	0.53
Number of chronic conditions ^a^, mean (SD)		4.0 (1.7)	3.3 (1.4)	0.02
Number of medications, mean (SD)		11.1 (5.9)	10.4 (6.0)	0.52
Documentation results				
Number of charts reviewed		*N* = 71 (baseline 25, follow-up 46)	*N* = 71 (baseline 25, follow-up 46)	
Overall prognosis	Baseline	1/25	0/25	>0.99
	Follow-up	8/46	0/46	0.006
Cancer screening	Baseline	15/25	13/25	0.78
	Follow-up	17/46	26/46	0.09
Overall prognosis documentation among charts that documented cancer screening	Baseline	0/15	0/13	>0.99
	Follow-up	3/17	0/26	0.06

^a^Out of 30 conditions in the Elixhauser comorbidity index [34]

In the intervention group, overall prognosis was documented in 1/25 charts (4 %) at baseline and 8/46 charts (17 %) at follow-up (*p* = 0.15). Five charts included quantitative estimates of prognosis; two cited a prognostic tool. Two charts documented prognosis communication: one as part of a goals of care discussion, another discussed how patient’s life expectancy might vary according to smoking status as part of smoking cessation counseling. Four charts documented prognosis affecting a clinical decision (e.g. *“recommendation is 7.5–8 % goal hemoglobin A1C for older adults with life expectancy <10 years”*). We found no documentation of overall prognosis in the control group at either time period. The difference between the two groups at follow-up (8/46 vs. 0/46) was significant (*p* = 0.006). We found cancer screening documentation in 32 charts in the intervention group (15 baseline, 17 follow-up) and 39 charts in the control group (13 baseline, 26 follow-up). Only three charts in the intervention group at follow-up documented both cancer screening and overall prognosis.

## Discussion

We describe the development and evaluation of an innovative curriculum that teaches internal medicine residents how to assess, communicate and apply prognosis to inform clinical decisions in the care of older adults with multimorbidity. This addresses a significant gap in the literature as we find no previously described curriculum that teaches about incorporating prognosis in the care of this patient population. While existing curricula teach communication or assessment of prognosis in oncology or palliative care [21–26], none addresses the unique challenges of incorporating prognosis in the care of older adults with multimorbidity. Without a predominant terminal illness such as cancer or being at the very end of life, older adults with multimorbidity may be less likely to consider prognosis relevant [35]. On the other hand, a growing literature suggests that life expectancy in the range of a few years impacts a number of clinical decisions [1, 4–9]. Our curriculum is innovative not only in the patient population it targets but also in teaching about a longer time frame of prognostication.

Although our curriculum uses many of the same strategies, such as case discussions, reflection, and role play, as other curricula that teach prognosis communication [21, 24–26], it is innovative in combining role play with subsequent guided experiential learning with actual patients in clinical practice. Experiential learning theory suggests that knowledge and skill gained via this form of learning are more substantive and long-lasting [30].

We found significant improvement in the intervention group’s knowledge and ability to use evidence-based tools to estimate prognosis. Although there is no study directly comparing the predictive accuracy of validated prognostic tools with clinical intuition, there are suggestions that clinicians’ intuition may be inaccurate and these tools may be helpful in clinical practice [20, 36–38].

We found an increase in documentation of overall prognosis. This did not reach statistical significance although Kolb’ model of experiential learning that was used in the curriculum was suggested to support long-lasting learning. Several factors may contribute to why rates of prognosis documentation only increased moderately in the intervention group. First, documentation may not accurately capture what occurred during a visit [39]. The resident may have considered the patient’s prognosis in clinical decisions without documenting it. Second, prognosis may not be discussed or documented in every visit; it may have been documented in a previous visit and be missed in the chart review. Without a gold standard to assess all the specific clinical scenarios in which prognosis should inform clinical decisions, we could not assess if prognosis should have been incorporated and documented in all of the visits included in chart review; prognosis may not have been relevant to the clinical decisions during a particular visit. Third, as our institution’s electronic medical record moves towards more information-sharing with patients, the residents may not have felt comfortable documenting prognosis without first discussing prognosis with the patient; and the residents may not have discussed prognosis with patients due to time constraints or that the patient was not ready to discuss prognosis [40]. Literature describes numerous barriers in effecting behavior change in clinicians [41]. In addition to this educational intervention, a multi-faceted approach that also targets the clinical practice environment and patients may be needed to achieve more significant changes.

This project has several limitations. As a novel intervention, this curriculum was implemented at one institution; the results may not reflect residents or patients elsewhere. Evaluation focused on short-term outcomes and we cannot draw conclusions about long-term impact of the curriculum. Additional evaluation in larger and different populations and of long-term outcomes are needed after curriculum dissemination. Second, although the two residency programs involved in the study are both affiliated with the same academic institution, the residents may not be completely comparable in relevant characteristics such as baseline geriatric training. However, both programs are highly selective academic residency programs and both groups of residents are first year trainees. There were no significant differences in the groups’ survey responses or chart review results at baseline. Third, there are no existing validated instruments to assess knowledge or behavior in incorporating prognosis. We developed our own survey instrument and chart review abstraction guide and tested both for face and content validity. Inter-rater agreement in chart review was high. However, further validity, reliability and psychometric testing of the instruments are needed. The multiple-choice question format used in part of knowledge assessment may have been susceptible to repeated testing bias and random error. Fourth, self-reported skill levels and documentation may not accurately reflect actual skill and behavior, respectively. We plan in a future study to evaluate residents’ communication skills through audio-recording the clinic visits, triangulate the information with resident survey and chart review to better capture practice patterns. Lastly, in the chart review, the patient populations served by the two study groups are different in gender and race. Limited by the low frequency of positive outcomes, we were not able to stratify the results by gender and race or adequately adjust for clustering of residents and patients, which may affect the accuracy of results.

## Conclusion

Older adults with multimorbidity are a heterogeneous population in which applying prognosis to inform clinical decisions is critical. Clinicians currently lack adequate training in this area. We describe a novel curriculum with significant short-term impact on prognosis knowledge and communication skills. This curriculum addresses an important educational gap in the care of older adults with multimorbidity.

## Additional file

Additional file 1:
**Incorporating Prognosis in the Care of Older Adults with Multimorbidity - Prognostic Resources.** (DOCX 834 kb)
