# Supplementary material for: Incorporating prognosis in the care of older adults with multimorbidity: description and evaluation of a novel curriculum
Source: BMC Med Educ. 2015 Dec 1;15:215. doi: 10.1186/s12909-015-0488-x (PMC4665923; doi:10.1186/s12909-015-0488-x)
Supplement: Additional file 1: — Incorporating Prognosis in the Care of Older Adults with Multimorbidity - Prognostic Resources. (DOCX 834 kb) [file 12909_2015_488_MOESM1_ESM.docx]

Incorporating Prognosis in the Care of Older Adults with Multimorbidity **-** Prognostic Resources

To assess life expectancy:

- If no single disease is predominantly severe → <http://eprognosis.org>
- If a single disease is predominantly severe → disease-specific indices
  - BODE index for COPD (<http://reference.medscape.com/calculator/bode-index-copd>)
  - Seattle heart failure model (<http://depts.washington.edu/shfm/index.php>)
  - Adjuvant for cancers (*Requires free registration: [https://www.adjuvantonline.com/](https://www.adjuvantonline.com/index.jsp))
  - ESRD:

[
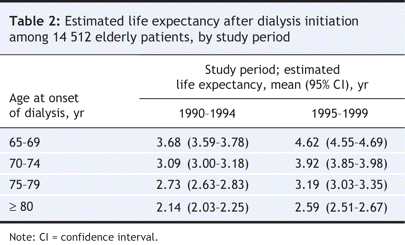
](http://www.cmaj.ca/content/177/9/1033/T2.large.jpg)

Jassal et al. CMAJ 177:1033-1038, 2007;

Another study showed people age 80s-90s has 1 year mortality rate of 46% after initiating HD (Kurella et al. Ann Intern Med 2007 Feb 6;146(3):177-83)

- If patient is at end of life
  - Hospice “end-stage indicators” (<http://www.montgomeryhospice.org/health-professionals/end-stage-indicators>)

| Dementia:   - dependent in bathing, dressing, continence, ambulation - no consistently meaningful verbal communication - at least 1: aspiration pneumonia, decubitus ulcer (multiple, stage 3-4), weight loss, UTI, sepsis fever |
| --- |
| Failure to thrive:   - BMI <22 and declining or not responding to artificial feeding. PPS ≤40% (see below) |
| End-stage heart disease:   - Optimally treated and still class IV HF (sx at rest) |
| End-stage liver disease:   - INR>1.5 and albumin <2.5; - At least 1: ascites (refractory or pt noncompliant), SBP, hepatorenal syndrome, hepatic encephalopathy (refractory or pt noncompliant), recurrent variceal bleeding |
| End-stage pulmonary disease:   - Significant SOB at rest - Progression (increased ED visits, hospitalizations, clinic visits) - Hypoxemia at rest on RA |
| Renal disease:   - If not seeking dialysis or transplant or discontinuing dialysis AND - Cr>8 (>6 for DM) OR CrCl <10 (<15 for DM) |

- - Palliative Performance Scale (mostly validated in cancer patients):

<https://www.capc.org/fast-facts/125-palliative-performance-scale-pps/>

To assess functional decline:

- Vulnerable elder scale: (<http://www.rand.org/content/dam/rand/www/external/health/projects/acove/docs/acove_ves13.pdf>)
- 5 year mortality (dotted line), mortality and functional decline (solid line)

Min, L. et al. (2009), The Vulnerable Elders-13 Survey Predicts 5-Year Functional Decline and Mortality Outcomes in Older Ambulatory Care Patients. Journal of the American Geriatrics Society, 57: 2070–2076.


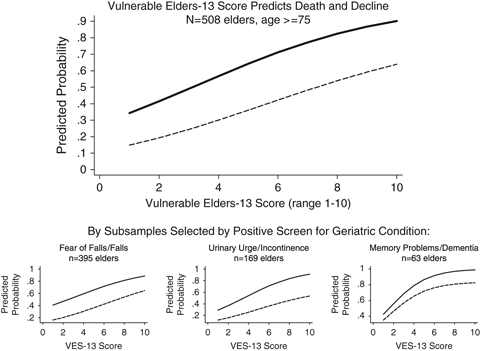


To assess condition specific prognosis/risk:

- Osteoporosis – FRAX calculator for fracture risk over 10 years: <http://www.shef.ac.uk/FRAX/>
- Atrial fibrillation:
  - Stroke risk: CHADS2-Vasc

<http://www.qxmd.com/calculate-online/cardiology/cha2ds2-vasc-stroke-risk-in-atrial-fibrillation>

- - Bleeding risk: HAS-BLED

<https://www.qxmd.com/calculate-online/cardiology/has-bled-score-bleeding-in-atrial-fibrillation>

- Diabetes – American Geriatrics Society Guidelines for care of **older adults** with DM / Choose Wisely Recommendation regarding A1C goals in older adults:
  - 7.0 – 7.5% in healthy older adults with long life expectancy
  - 7.5 – 8.0% in those with moderate comorbidity and a life expectancy < 10 years
  - 8.0 – 9.0% in those with multiple morbidities and shorter life expectancy

Guidelines abstracted from the american geriatrics society guidelines for improving the care of older adults with diabetes mellitus: 2013 update. [J Am Geriatr Soc.](http://www.ncbi.nlm.nih.gov/pubmed/?term=Guidelines+Abstracted+from+the+American+Geriatrics+Society+Guidelines+for+Improving+the+Care+of+Older+Adults+with+Diabetes+Mellitus%3A+2013+Update+%28JAGS+61%3A2020-2026%2C+2013%29) 2013 Nov;61(11):2020-6.

- Cancer screening:
  - ESRD Choosing Wisely from American Society of Nephrology

“Don’t perform routine cancer screening for dialysis patients with limited life expectancies,…such as those who are not transplant candidates, without signs or symptoms.”

Williams AW, Dwyer AC, Eddy AA, et al. Critical and honest conversations: The evidence behind the "choosing wisely" campaign recommendations by the american society of nephrology. *Clin J Am Soc Nephrol*. 2012;7(10):1664-1672.

- - Choosing Wisely from Society of General Internal Medicine

**“Don’t recommend cancer screening in adults with life expectancy of less than 10 years.”**

Society of General Internal Medicine. Five things physicians and patients should question - the choosing wisely campaign. 2013. <http://www.choosingwisely.org/wp-content/uploads/2013/09/SGIM-5things-List_091213.pdf>.

- Helpful tables on baseline risk of cancer-related mortality, and number needed to screen:

Walter, L. et al. Cancer Screening in Elderly Patients:  A Framework for Individualized Decision Making. JAMA. 2001;285(21):2750-2756.


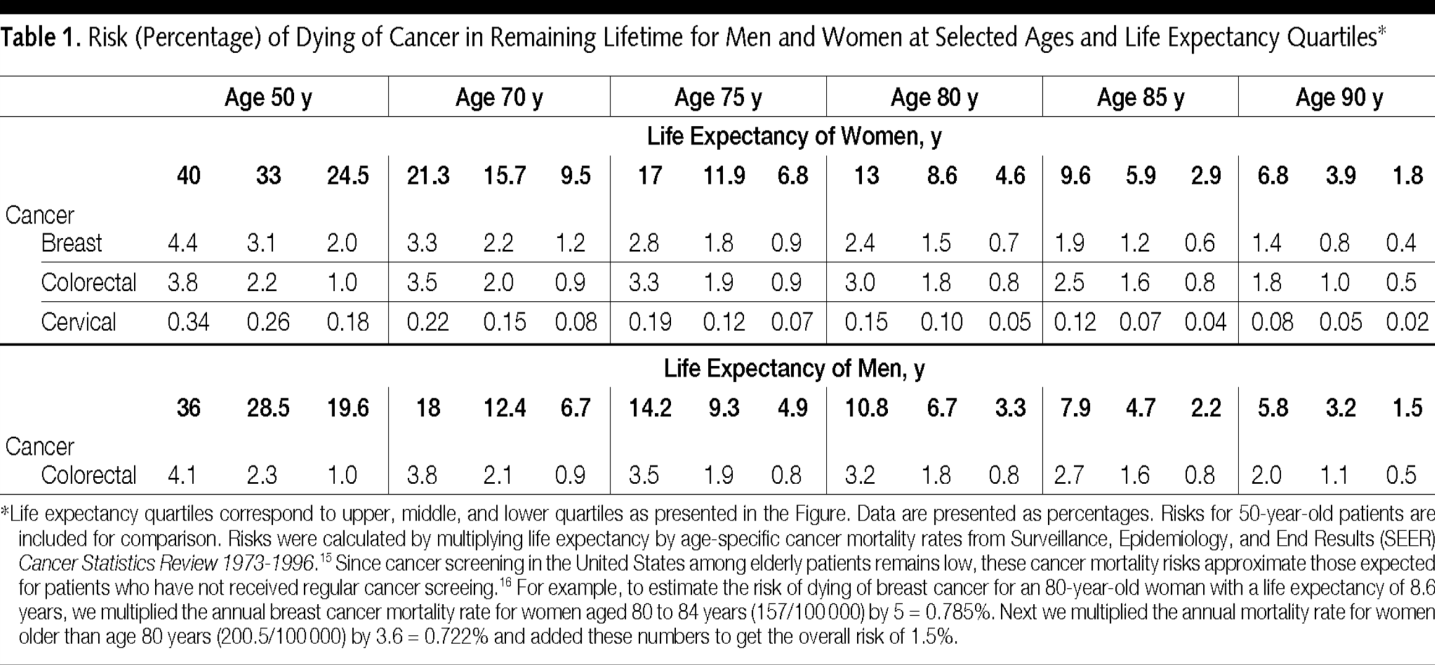


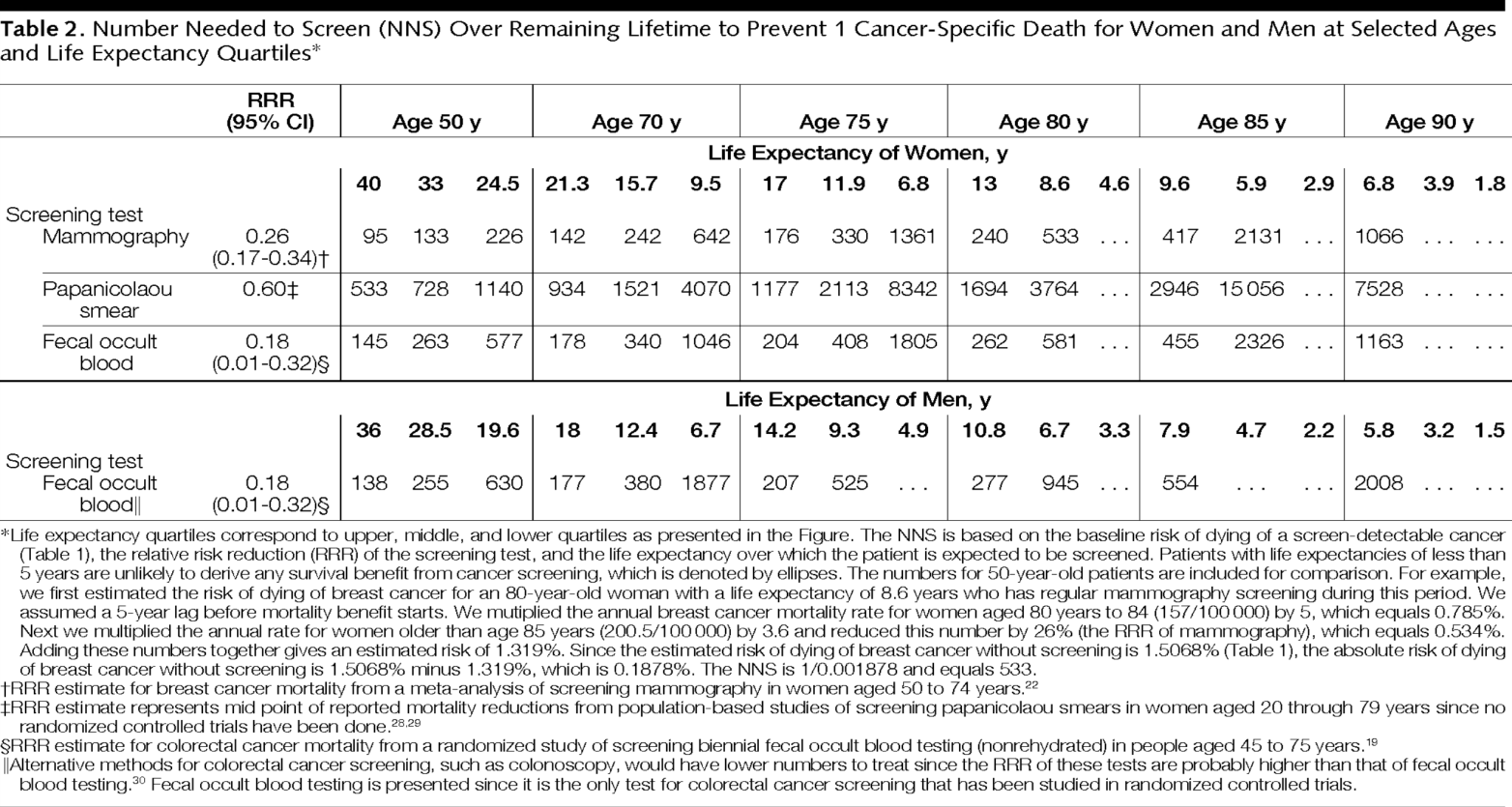


Time to benefit in breast and colorectal cancer screening – most appropriate for patients with life expectancy >10 years. On average, it took **10.7 years** (4.4 to 21.6) before one death from breast cancer was prevented for 1000 women screened, **10.3 years** (6.0 to 16.4) before one death from colorectal cancer was prevented for 1000 patients screened.

Lee SJ, et al. Time lag to benefit after screening for breast and colorectal cancer: Meta-analysis of survival data from the united states, sweden, united kingdom, and denmark. *BMJ*. 2013;346:e8441.


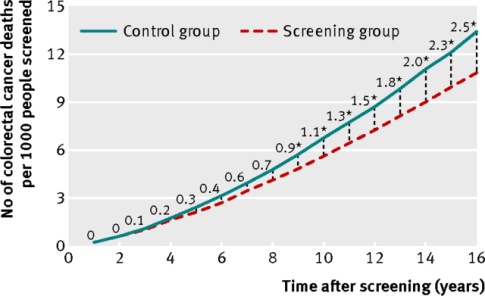

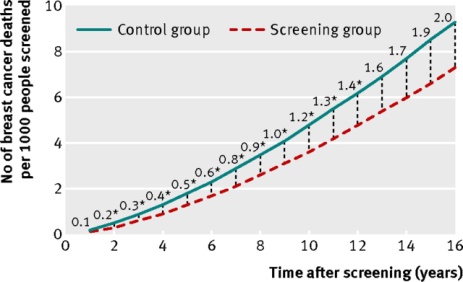


.
